# Differences in Multimodal Electroencephalogram and Clinical Correlations Between Early-Onset Alzheimer’s Disease and Frontotemporal Dementia

**DOI:** 10.3389/fnins.2021.687053

**Published:** 2021-08-05

**Authors:** Nan Lin, Jing Gao, Chenhui Mao, Heyang Sun, Qiang Lu, Liying Cui

**Affiliations:** Department of Neurology, Peking Union Medical College Hospital, Beijing, China

**Keywords:** EEG microstate, early onset Alzheimer’s disease, frontotemporal dementia, CSF biomarkers, spectral analysis

## Abstract

**Background:**

Alzheimer’s disease (AD) and frontotemporal dementia (FTD) are the two main types of dementia. We investigated the electroencephalogram (EEG) difference and clinical correlation in early-onset Alzheimer’s disease (EOAD), and FTD using multimodal EEG analyses. EOAD had more severe EEG abnormalities than late-onset AD (LOAD). Group comparisons between EOAD and LOAD were also performed.

**Methods:**

Thirty patients diagnosed with EOAD, nine patients with LOAD, and 14 patients with FTD (≤65 y) were recruited (2008.1–2020.2), along with 24 healthy controls (≤65 y, *n* = 18; >65 y, *n* = 6). Clinical data were reviewed. Visual EEG, EEG microstate, and spectral analyses were performed.

**Results:**

Compared to controls, markedly increased mean microstate duration, reduced mean occurrence, and reduced global field power (GFP) peaks per second were observed in EOAD and FTD. We found increased durations of class B in EOAD and class A in FTD. EOAD had reduced occurrences in classes A, B, and C, while only class C occurrence was reduced in FTD. The visual EEG results did not differ between AD and FTD. Microstate B showed correlations with activities of daily living score (*r* = 0.780, *p* = 0.008) and cerebrospinal fluid (CSF) Aβ42 (*r* = −0.833, *p* = 0.010) in EOAD. Microstate D occurrence was correlated with the CSF Aβ42 level in FTD (*r* = 0.786, *p* = 0.021). Spectral analysis revealed a general slowing EEG, which may contribute to microstate dynamic loss. Power in delta was significantly higher in EOAD than in FTD all over the head. In addition, EOAD had a marked increased duration and decreased occurrence than late-onset AD (LOAD), with no group differences in visual EEG results.

**Conclusion:**

The current study found that EOAD and FTD had different EEG changes, and microstate had an association with clinical severity and CSF biomarkers. EEG microstate is more sensitive than visual EEG and may be useful for the differentiation between AD and FTD. The observations support that EEG can be a potential biomarker for the diagnosis and assessment of early-onset dementias.

## Introduction

Alzheimer’s disease (AD) is the most common form of dementia, accounting for 60–80% of cases (Scheltens et al., 2016). A diagnosis of AD below the age of 65 is classed as early-onset AD (EOAD). EOAD accounts for only 5%–10% of all AD cases (Dai et al., 2018). Accumulation of abnormally folded amyloid beta (Aβ) and hyperphosphorylated tau proteins in amyloid plaques and neural tangles is causally related to neurodegenerative processes (Karran et al., 2011). Low Aβ_42_ levels, high concentrations of t-tau and p-ta, and the ratio of tau/Aβ_42_ help to discriminate AD from healthy controls and other dementias (Shaw et al., 2009; Casoli et al., 2019). Patients with EOAD display greater cerebrospinal fluid (CSF) anomalies (Dumurgier et al., 2013) and more severe electroencephalogram (EEG) abnormalities (Micanovic and Pal, 2014).

Frontotemporal dementia (FTD) accounts for approximately 10% of all dementias (Hogan et al., 2016), characterized by prominent changes in social behavior and personality or aphasia accompanied by pathological changes in the frontal and temporal lobes. TAR (*trans-*active response) DNA-binding protein 43, tau, and fused-in-sarcoma protein were the three major disease proteins in the neuropathology of FTD (Wang et al., 2013). It can be difficult to distinguish clinically FTD from AD, especially EOAD, as EOAD may more commonly manifest with non-memory presentations, like language problems (Koedam et al., 2010).

Electroencephalogram is a relatively cost-effective, non-invasive technique, increasingly considered to be a potential biomarker for dementia differentiation recently. Several characteristics of the EEG have been put forward as biomarkers in AD and might be useful in the early recognition of neural signatures of dementias and differential diagnosis. Spectral EEG measures in AD showed a reduction of alpha and beta spectral powers and an increase in theta and delta spectral powers. The changes were associated with disease severity (Horvath et al., 2018). Recently, EEG microstate analyses were used in dementia. EEG microstates are defined as quasi-stable brief patterns of coordinated electrical activity on the scalp surface, which was first described by Lehmann et al. (1987) (Schumacher et al., 2019). The topographies remained transiently stable for 60–150 ms before rapidly transitioning into a new state.

Electroencephalogram microstates have been shown to be associated with cognition and perception (Milz et al., 2016; Santarnecchi et al., 2017). Previous studies observed microstate changes in cognitive disorders (Stevens and Kircher, 1998; Nishida et al., 2013; Hatz et al., 2015; Musaeus et al., 2019; Schumacher et al., 2019; Smailovic et al., 2019; Tait et al., 2020). However, microstate characteristics and correlation with CSF biomarkers in early-onset dementias, including AD and FTD, have not been well studied. The current study was set to investigate the EEG microstate in EOAD and FTD, along with EEG spectral analysis, and the correlations with clinical data and CSF biomarkers. The differences in EEG data were then analyzed to test the utility of EEG as a biomarker for clinical evaluations and differential diagnosis. Comparisons between EOAD, late-onset AD (LOAD), and healthy controls were also performed to investigate the difference between microstate and visual EEG and the effect of age.

## Materials and Methods

### Patients

The study population consisted of patients with cognition impairment in Peking Union Medical College Hospital between June 2015 and October 2019. Patients were diagnosed based on information obtained from an extensive clinical history, physical examinations, and lab examinations and excluded mood disorders and schizophrenia. All patients diagnosed with AD met the IWG-II criteria (Dubois et al., 2014) with cognitive scales, brain MRI, and CSF biomarker results. For FTD diagnosis, the Neary et al. (1998) or the McKhann et al. (2001) criteria were employed with brain MRI and CSF biomarker results for differentiation from AD. Dementia diagnoses were performed independently by two experienced clinicians. Patients who had complications of other neurological or psychiatric disorders, and severe systemic diseases that may influence the central nervous system, were excluded. Patients with AD were divided into EOAD and LOAD by age 65. Patients with FTD who were older than 65 years were further excluded. Clinical assessment scales included the Mini-Mental State Examination (MMSE) (Folstein et al., 1975), the Montreal Cognitive Assessment (MoCA) (Nasreddine et al., 2005), and activities of daily living (ADL) score. The data of CSF biomarkers and cognitive assessments undergone at the same time with EEG recordings were used for further analyses.

### Biomarkers Assessments

Cerebrospinal fluid t-tau, p-tau, and Aβ_42_ were measured using an enzyme-linked immunosorbent assay (Fujirebio, Ghent, Belgium). Samples were handled by experienced senior laboratory technicians blinded to patients’ information.

### EEG Examination and Data Preprocessing

Video EEG monitoring was performed using a 19-channel video-EEG monitoring system (EEG-1200C, Nihon Kohden, Tokyo, Japan) in hospital for more than 2 h. Recording electrodes were placed according to the international 10–20 system with a sampling frequency of 500 Hz. The visual EEG results were evaluated by at least one experienced epilepsy specialist. The degree of visual EEG abnormality was scored as follows: (1) 0 = normal; (2) 1 = mildly abnormal; (3) 2 = moderately abnormal; and (4) 3 = severely abnormal (Table 1).

**TABLE 1:** Definitions of visual EEG abnormality scores.

Visual EEG scores	Definitions
1 = mildly abnormal	At least one of the following EEG patterns: •<50% asymmetrical background activity; • Irregular alpha rhythm; • Excess beta activity with amplitude >50 μV, • Excessive theta activity mainly over the frontal region • Mildly excessive delta activity
2 = moderately abnormal	At least one of the following EEG patterns: • Occipital 7–8-Hz frequency band • No obvious occipital alpha rhythm • Asymmetry [>50%] moderately high delta activities • Sporadic epileptiform discharges
3 = severely abnormal	At least one of the following EEG patterns: • Persistent low-voltage or electrical silence • Periodic phenomenon • Dominant background delta or theta activity • Rhythmic epileptiform discharges

Resting-state EEG data without excessive noise, artifacts, and epileptiform discharges were preprocessed with EEGLAB (R13_6_5b) in MATLAB R2017a. An independent component analysis was used for further artifact removal. Data were bandpass filtered into the range of 0.1–40 Hz and were recomputed against the average reference. EEG data were split into non-overlapping epochs of 2 s. Patients with less than 25 epochs were excluded. It resulted in 30 patients with EOAD, 14 with FTD, and 18 healthy controls (≤65 y) for further analyses. Moreover, nine patients with LOAD and six healthy controls (>65 y) were included in the current study. Frequency spectral analysis was performed in the following frequency bands: delta (1–4 Hz), theta (4–8 Hz), alpha (8–12 Hz), and beta (12–30 Hz).

### Microstate Analysis

The microstate analysis was conducted using the EEGLAB plugin Microstate 1.1 in MATLAB R2017a. EEG data were further bandpass filtered into the 1–20-Hz range for microstate analysis. The overall variances across all electrodes were quantified by measuring the global field power (GFP). GFP was calculated as the standard deviation of the data at each time point (Wackermann et al., 1993; Hatz et al., 2015):

$$G\,F\,P_{t}=\sqrt{\frac{\sum_{i=1}^{n}u_{i}^{2}}{n}}$$

(n=numberofchannels

u=amplitudeinuVattimepointt)

Electroencephalogram topographies tend to be stable during periods of high GFP (Lehmann et al., 1987). The scalp maps at the momentary peaks of the GFP were extracted and clustered using a k-means cluster analysis (Hatz et al., 2015). Previous studies revealed that the optimal number of microstate classes belonged to two to six classes (mean 3.7 classes), according to the agglomerative clustering procedure (Lehmann et al., 1993; Khanna et al., 2015; Michel and Koenig, 2018). The current study used a cross-validation criterion and the Krzanowski–Lai criterion by the Cartool software (Brunet et al., 2011) to determine the optimal number of microstate classes, testing the entire range of 1–12 classes.

The cluster analysis resulted in mean microstate topographies for each class. Each group model maps were created based on individual model maps. The resulting class-labeled group microstate maps were then fit back to the templates to assign model maps to each participant.

Microstate topographies of each microstate class were compared between groups using a non-parametric randomization test (TANOVA, topographical analysis of variance), as implemented in the Ragu software (Koenig et al., 2011). GFP peaks per second (PPS), microstate duration (ms), frequency of occurrence of each microstate (/s), the percentage of total analysis time covered by each microstate (%), and transition probabilities were calculated.

### Frequency Spectral Analysis

Frequency spectral analysis was performed using a fast Fourier transform (FFT, 1,000-point) algorithm. The absolute power spectral density [PSD, dB, 10 log_10_(V^2^/Hz)] for each channel based on the periodogram was calculated. The relative PSD (rPSD) was computed by normalizing the total power in the whole frequency range. The absolute and relative PSDs were averaged across channels within groups to measure global comparisons between groups in each frequency band.

### Statistics Analyses

The relatively symmetrical data distribution of microstate, rPSD, and absolute PSD is shown in the box plots (Supplementary Material 1). Although there were outliers, it intuitively conformed to the normal assumption. Multivariate analysis of variance (MANOVA) was therefore performed to assess group differences of microstate variables. When overall significant effects were found, univariate ANOVAs followed by *post hoc* analyses with Bonferroni correction were performed. A Spearman correlation test was used for the correlation analysis. Continuous non-normal data were examined using the Kruskal–Wallis test or Mann–Whitney *U* test for group comparisons. The chi-square test was used for group comparison of categorical data. The level of significance was set at 0.05. Statistics analyses were performed using IBM SPSS Statistics v22.

## Results

Clinical and demographic data between dementia and control groups are presented in Table 2. Controls, EOAD, and FTD participants had no significant differences in age and gender. The FTD group was significantly less impaired in the MMSE than EOAD group (*p* = 0.015). Additionally, the two dementia groups did not differ significantly in terms of dementia duration, ADL, and MoCA. The percentage of patients taking acetylcholinesterase inhibitors (AChEIs) at the same time of EEG recordings did not differ between the two dementia groups.

**TABLE 2:** Demographic, clinical data, and CSF biomarkers in dementia and control groups.

	HC (*n* = 18)	EOAD (*n* = 30)	FTD (*n* = 14)	*p*
Age (mean, range)	54 (44–64)	55 (41–64)	57 (47–64)	*p* = 0.112
Gender (M:F)	10:8	11:19	8:6	*p* = 0.476
Disease course (mean, range, /y)	–	3.4 (0.25–13)	3.1 (0.75–6)	*P*_*AD–FTD*_ = 0.603
AChEI	–	16 (53.3%)	5 (35.7%)	*P*_*AD–FTD*_ = 0.342
MMSE (mean, range	–	12 (1–25, *n* = 29)	19 (6–27, *n* = 9)	*P*_*AD–FTD*_ = 0.010
MoCA (mean, range)	–	13 (8–21, *n* = 7)	17 (12–19, *n* = 6)	*P*_*AD–FTD*_ = 0.234
ADL (mean, range)	–	33 (20–50, *n* = 10)	25 (0–39, *n* = 6)	*P*_*AD–FTD*_ = 0.313
CSF biomarkers (pg/ml, mean, range)	–	*n* = 8	*n* = 8	
Aβ_42_		420 (281–550)	658 (287–870)	*P*_*AD–FTD*_ = 0.065
T-tau		445 (94–1573)	261 (117–587)	*P*_*AD–FTD*_ = 0.505
P-tau		69.2 (40.9–122)	47.3 (26.7–71)	*P*_*AD–FTD*_ = 0.13
T-tau/Aβ_42_		1.18 (0.20–4.59)	0.46 (0.15–1.09)	*P*_*AD–FTD*_ = 0.083
P-tau/Aβ_42_		0.18 (0.11–0.36)	0.09 (0.03–0.20)	*P*_*AD–FTD*_ = 0.028
APOE ε4	–			*P*_*AD–FTD*_ = 0.782
0		7/11 (63.6%)	4/7 (57.1%)	
1		3/11 (27.3%)	3/7 (42.9%)	
2		1/11 (9.1%)	0	
Visual EEG score				*P*_*AD–FTD*_ = 0.304
0	24 (100%)	13 (43.3%)	8 (57.1%)	
1		3 (10.0%)	2 (14.3%)	
2		14 (46.7%)	4 (28.6%)	
3		0	0	

*HC, healthy controls; EOAD, early-onset Alzheimer disease; FTD, frontotemporal dementia.*

Cerebrospinal fluid biomarker results were available in eight patients with EOAD and eight with FTD at the time of EEG recordings. The levels of Aβ_42_, t-tau, and p-tau did not differ between the EOAD and FTD groups. However, the ratio of p-tau to Aβ_42_ was shown to be significantly higher in EOAD, compared to FTD (*p* = 0.028). A percentage of 36.4% (4/11) subjects in the EOAD group were APOE ε4 carriers, of which one patient (11.1%) had two copies. For the FTD group, 42.9% (3/7) of patients were carriers of the APOE ε4 genotype, and no one had APOE ε4 homozygotes.

Overall, 17 EOAD patients had abnormal EEG results, including three patients with scores of 1, and 14 with scores of 2. For the FTD group, eight patients had scores of 0, two had scores of 1, and four had scores of 2. No participants had severe abnormal EEG results. The main EEG visual signs were abnormal or disappeared posterior dominant alpha rhythm and anterior dominant or diffuse slowing. Only one patient with EOAD had epileptiform discharges. The Mann–Whitney *U* test showed no significant group difference in visual EEG severity.

### EEG Microstates

The median optimal number of microstate classes in the EOAD and control groups was four, while the median in FTD was five. The overall median optimal number in the entire dataset was four. Therefore, the number of microstate classes was therefore set to four for further analyses, commonly used in most studies, labeled as A, B, C, and D (Koenig et al., 1999; Michel and Koenig, 2018). The mean global explained variance (standard deviation, SD) of four microstates in each group was 79.8% (3.3%) for controls, 74.1% (2.3%) for EOAD, and 77.0% (4.4%) for FTD.

**FIGURE 1:**
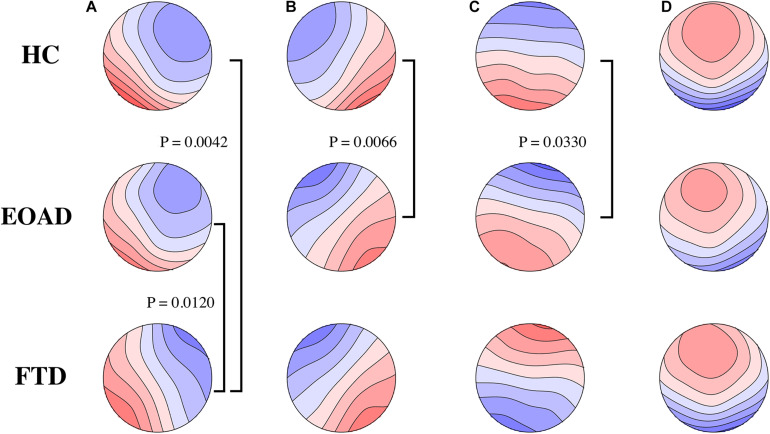
Microstate class topographies. The letters of **(A–D)** on the top represent the four microstate classes, respectively. Group comparisons used by TANOVA. Significant *p*-values after Bonferroni correction are illustrated. FTD had different microstate **(A)** map from HC and EOAD. Microstate **(B,C)** map was different in EOAD, compared to HC. No group difference in microstate **(D)** was observed. FTD, frontotemporal dementia; EOAD, early onset Alzheimer’s disease; HC, healthy controls.

Group microstate maps are illustrated in Figure 1. After application of the Bonferroni correction, TANOVAs for each microstate class showed that the EOAD maps were different from control maps for classes B and C, and FTD maps were different from EOAD and control maps for class A. There were no significant group differences between FTD and controls in model map topography for classes B, C, and D.

**FIGURE 2:**
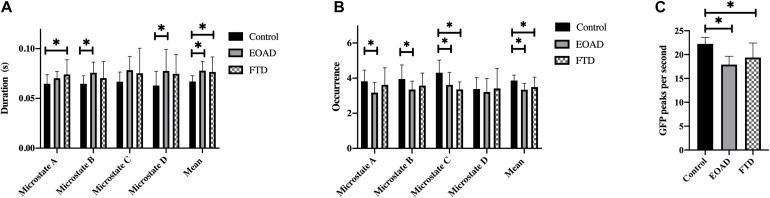
Microstate characteristics. Group comparison of microstate duration **(A)**, occurrence **(B)**, and GFP peaks per second **(C)**. *p*-values result from pairwise *post hoc* tests following univariate ANOVAs. ^∗^*p* < 0.05. FTD, frontotemporal dementia; EOAD, early-onset Alzheimer’s disease; HC, healthy controls; GFP, global field power.

Across all microstate classes, the mean microstate duration was 66.9 ms in controls, 77.8 ms in EOAD patients, and 76.6 ms in FTD patients. The mean duration in dementia groups was increased significantly compared to controls (*P*_*HC–EOAD*_ = 0.002; *P*_*HC–FTD*_ = 0.028) (Table 3). The mean number of unique microstate occurrences per second and PPS was reduced in EOAD and FTD, compared to HC (mean occurrence: *P*_*HC–EOAD*_ < 0.001, *P*_*HC–FTD*_ = 0.035; PPS: *P*_*HC–EOAD*_ < 0.001, *P*_*HC–FTD*_ = 0.001) (Figure 2).

**TABLE 3:** EEG microstate data in dementia and control groups.

Duration/ms (Std)	HC (*n* = 18)	EOAD (*n* = 30)	FTD (*n* = 14)	ANOVA (2,59)	P_*HC–EOAD*_	P_*EOAD–FTD*_	P_*HC–FTD*_
A	64.7 (9.1)	70.3 (6.9)	74.1 (14.8)	*F* = 3.805 *p* = 0.028	0.181	0.703	0.028
B	64.6 (8.1)	75.7 (10.7)	70.4 (16.6)	*F* = 5.140 *p* = 0.009	0.007	0.493	0.510
C	66.8 (9.7)	78.3 (13.9)	75.3 (25.2)	*F* = 2.913 *p* = 0.062			
D	63.0 (14.3)	77.5 (21.8)	74.6 (19.5)	*F* = 3.279 *p* = 0.045	0.043	1.000	0.290
Mean duration	66.9 (5.8)	77.8 (9.3)	76.6 (15.0)	*F* = 7.008 *p* = 0.002	0.002	1.000	0.028

**Occurrence/s (Std)**	**HC**	**EOAD**	**FTD**	**ANOVA (2,59)**	**P_*HC–EOAD*_**	**P_*EOAD–FTD*_**	**P_*HC–FTD*_**

A	3.82 (0.64)	3.18 (0.57)	3.62 (0.97)	*F* = 5.219 *p* = 0.008	0.009	0.167	1.000
B	3.95 (0.79)	3.35 (0.47)	3.57 (0.72)	*F* = 4.981 *p* = 0.010	0.008	0.889	0.292
C	4.31 (0.72)	3.62 (0.71)	3.36 (0.42)	*F* = 9.439 *p* ≤ 0.001	0.003	0.682	<0.001
D	3.38 (0.65)	3.21 (0.76)	3.42 (1.14)	*F* = 0.391 *p* = 0.678			
Mean occurrence	3.87 (0.31)	3.34 (0.36)	3.49 (0.57)	*F* = 9.757 *p* ≤ 0.001	<0.001	0.724	0.035
PPS	22 (1.4)	18 (1.7)	19 (3.0)	*F* = 25.879 *p* ≤ 0.001	<0.001	0.085	0.001

*HC, healthy controls; EOAD, early-onset Alzheimer disease; FTD, frontotemporal dementia; PPS, global field power peaks per second.*

Microstate analysis results are presented in Table 3 and Figure 2. There were no significant differences between the EOAD and FTD groups. Compared to controls, microstate A duration in FTD and microstate B and D durations in EOAD were increased. Microstate C occurrence was reduced in both dementia groups compared to controls, with no significant difference between EOAD and FTD groups. Microstate A and B occurrences were significantly reduced in EOAD, compared to controls. No significant group differences were observed in microstate coverage and transition probabilities (Supplementary Material 2).

### Relation Between Microstate and Clinical/CSF Biomarker Data

We found that the degree of visual EEG abnormality was negatively correlated with MMSE score (*r* = −0.380, *p* = 0.042) in EOAD. Visual EEG scores were positively correlated with disease course (*r* = 0.631, *p* = 0.021), p-tau (*r* = 0.756, *p* = 0.030), and the ratio of t-tau to Aβ_42_ (*r* = 0.756, *p* = 0.030) in FTD.

In the EOAD group, microstate B coverage was negatively correlated with the concentration of CSF Aβ_42_ (*r* = −0.833, *p* = 0.010) and was positively correlated with the ADL score (*r* = 0.780, *p* = 0.008). Additionally, the transition probability from A to B was positively related to the ADL score (*r* = 0.657, *p* = 0.039) and negatively related to the CSF Aβ_42_ concentration (*r* = −0.714, *p* = 0.047). The P-tau concentration was negatively related to the transition probability from A to C (*r* = −0.738, *p* = 0.037).

In the FTD group, the CSF Aβ_42_ level was positively related to microstate D occurrence (*r* = 0.786, *p* = 0.021) and transition probability from D to A (*r* = 0.714, *p* = 0.047). There was a negative correlation between the mean occurrence and CSF t-tau concentration (*r* = −0.714, *p* = 0.047). ADL was negatively related to transition probability from D to B (*r* = −0.886, *p* = 0.019).

**FIGURE 3:**
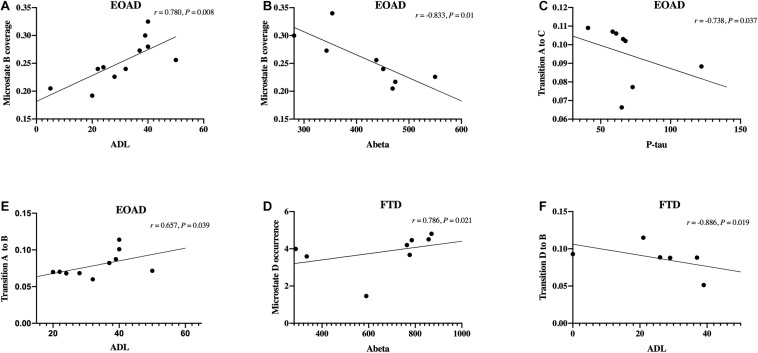
Correlations between microstate and clinical data. **(A–F)** Spearman’s correlations between microstate variables and clinical data, including cognitive scores and cerebrospinal fluid biomarkers levels. FTD, frontotemporal dementia; EOAD, early-onset Alzheimer’s disease; ADL, activities of daily living.

The microstate variables were not significantly correlated with MoCA scores, ratios of p-tau to Aβ_42_, and the number of APOE ε4copies in both groups. PPS showed no correlation with cognitive scores and CSF biomarkers. The Spearman correlations with a relatively high significance level (*p* < 0.040) are illustrated in Figure 3. The correlations with *p*-value > 0.040 required a larger sample to be confirmed.

### EEG Microstate in Early- and Late-Onset AD

Group comparisons in EOAD (*n* = 30), LOAD (*n* = 9), and age-matched controls (young: *n* = 18, old: *n* = 6) were performed. Demographic data and clinical assessment scales are presented in Table 4. There are no significant differences between the AD subgroups in terms of gender, MMSE, MoCA, ADL, and visual EEG score.

**TABLE 4:** Clinical and EEG microstate data in early- and late-onset AD and age-matched controls.

	HC		AD		
		
	Younger (1) *n* = 18	Older (2) *n* = 6	EOAD (3) *n* = 30	LOAD (4) *n* = 9	
Age (mean, range)	54 (44–64)	71 (68–74)	55 (41–64)	69 (65–75)	
Gender (M:F)	10:8	4:2	11:19	5:4	*P*_*EOAD–LOAD*_ = 0.398
Disease course (mean, range, /y)			3.4 (0.25–13.0)	3.0 (1.0–6.0)	*P*_*EOAD–LOAD*_ = 0.844
AChEI			16:14	3:6	*P*_*EOAD–LOAD*_ = 0.451
MMSE (mean, range			12 (1–25)	14 (3–27)	*P*_*EOAD–LOAD*_ = 0.493
MoCA (mean, range)			13 (8–21)	11 (6–15)	*P*_*EOAD–LOAD*_ = 0.831
ADL (mean, range)			33 (20–50)	35 (22–54)	*P*_*EOAD–LOAD*_ = 0.524
Visual EEG score			0 (*n* = 13) 1 (*n* = 3) 2 (*n* = 14)	0 (*n* = 5) 1 (*n* = 2) 2 (*n* = 2)	*P*_*EOAD–LOAD*_ = 0.327

**Duration/ms (Std)**	**1**	**2**	**3**	**4**	**ANOVA (3,59)**

A	64.7 (9.1)	64.9 (6.7)	70.3 (6.9)	64.9 (6.7)	*F* = 2.913 *p* = 0.042
B	64.6 (8.1)	66.45 (5.3)	75.7 (10.7)	64.9 (10.6)	*F* = 7.042 *p* < 0.001 *P*_1–3_ = 0.001, *P*_3–4_ = 0.024
C	66.8 (9.7)	86.0 (28.6)	78.3 (13.9)	63.5 (10.5)	*F* = 3.322 *p* = 0.026
D	63.0 (14.3)	64.8 (17.9)	77.5 (21.8)	65.7 (12.5)	*F* = 2.999 *p* = 0.038 *P*_1–3_ = 0.043
Mean duration	66.9 (5.8)	74.1 (14.4)	77.8 (9.3)	66.0 (7.9)	*F* = 7.401 *p* < 0.001 *P*_1–3_ = 0.001, *P*_3–4_ = 0.005

**Occurrence/s (Std)**	**1**	**2**	**3**	**4**	**ANOVA (3,59)**

A	3.82 (0.64)	3.52 (1.23)	3.18 (0.57)	4.19 (0.73)	*F* = 6.899 *p* < 0.001 *P*_1–3_ = 0.008, *P*_3–4_ = 0.002
B	3.95 (0.79)	3.72 (0.96)	3.35 (0.47)	3.99 (0.43)	*F* = 4.316 *p* = 0.008 *P*_1–3_ = 0.032
C	4.31 (0.72)	4.47 (0.54)	3.62 (0.71)	3.79 (0.70)	*F* = 4.961 *p* = 0.004 *P*_1–3_ = 0.005
D	3.38 (0.65)	2.75 (0.38)	3.21 (0.76)	3.75 (0.59)	*F* = 1.746 *p* = 0.167
Mean occurrence	3.87 (0.31)	3.61 (0.59)	3.34 (0.36)	3.93 (0.46)	*F* = 9.435 *p* < 0.001 *P*_1–3_ < 0.001, *P*_3–4_ = 0.001

**Coverage (Std)**	**1**	**2**	**3**	**4**	**ANOVA (3,59)**

A	0.25 (0.06)	0.22 (0.07)	0.22 (0.04)	0.26 (0.04)	*F* = 3.2 *p* = 0.030
B	0.25 (0.07)	0.25 (0.06)	0.25 (0.04)	0.25 (0.04)	*F* = 0.16 *p* = 0.923
C	0.28 (0.07)	0.35 (0.07)	0.28 (0.07)	0.24 (0.05)	*F* = 1.867 *p* = 0.145
D	0.22 (0.08)	0.18 (0.06)	0.25 (0.11)	0.24 (0.06)	*F* = 0.999 *p* = 0.400
PPS (Std)	22 (1.4)	21 (2.7)	18 (1.7)	21 (2.6)	*F* = 21.441 *p* < 0.001 *P*_1–3_ < 0.001, *P*_3–4_ = 0.003

*HC, healthy controls; EOAD, early-onset Alzheimer disease; LOAD, late-onset Alzheimer disease; PPS, global field power peaks per second.*

The differences between HC and EOAD groups were consistent with the above observations. Moreover, we found that microstate B duration and the mean duration were significantly increased in EOAD, compared with LOAD. Microstate A occurrence, the mean occurrence, and PPS were reduced in EOAD, compared with LOAD. Additionally, a preferential transition to microstates A from D was revealed in LOAD, compared to age matched controls (0.090 vs. 0.047, *p* = 0.02). No significant group differences were observed in LOAD and age-matched controls in other microstate analyses.

### Frequency Spectral Analysis

**FIGURE 4:**
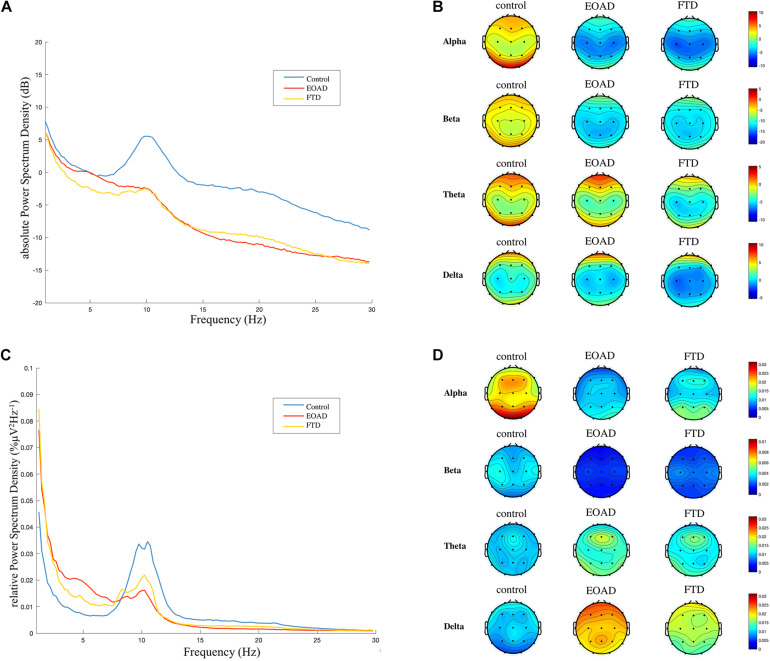
Frequency spectral analysis. **(A)** and **(C)** Across-channel grand average of absolute power spectral density (PSD, dB) and relative PSD (%μV^2^Hz^–1^) over frequency for each group are illustrated. **(B)** and **(D)** The topographies were calculated from PSD in each frequency band. A general slowing EEG was presented in both FTD and EOAD groups. FTD, frontotemporal dementia; EOAD, early-onset Alzheimer’s disease; HC, healthy controls.

The across-channel grand average of global EEG PSD in each group is illustrated in Figures 4A,C. The means of the absolute PSD in the control group were higher than in the dementia groups in alpha and beta bands with significance (Figure 4A and Table 5). As shown in Figure 4C and Table 5, the global rPSD in dementia groups was significantly reduced in alpha and beta bands and increased in delta bands, compared to controls. Moreover, rPSD in the theta band was higher in EOAD, compared to the control group. The topographies calculated from the global absolute and relative PSDs over frequency bands were illustrated in Figures 4B,D. The topographies revealed that PSD changes were presented in the whole scalp regions.

**TABLE 5:** Power spectral density in dementia and control groups.

PSD (dB) (mean, std)	HC *n* = 18	EOAD *n* = 30	FTD *n* = 14	MANOVA (2, 59)	P_*HC–EOAD*_	P_*EOAD–FTD*_	P_*HC–FTD*_
1–4 Hz	2.90 (8.1)	1.91 (2.88)	0.74 (3.32)	*F* = 0.716 *p* = 0.493			
4–8 Hz	−0.07 (8.54)	−0.91 (2.72)	−2.90 (2.98)	*F* = 1.231 *p* = 0.299			
8–12 Hz	3.73 (8.65)	−3.21 (3.69)	−3.53 (3.81)	*F* = 10.085 *p* ≤ 0.001	<0.001	1.000	0.002
12–30 Hz	−4.15 (8.38)	−11.19 (2.40)	−10.73 (2.54)	*F* = 12.445 *p* ≤ 0.001	<0.001	1.000	0.001

**rPSD (%, mean, std)**	**HC**	**EOAD**	**FTD**	**MANOVA (2,59)**	**P_*HC–EOAD*_**	**P_*EOAD–FTD*_**	**P_*HC–FTD*_**

1–4 Hz	0.98 (0.4)	2.26 (0.54)	1.70 (0.81)	*F* = 25.725 *p* ≤ 0.001	<0.001	0.025	0.003
4–8 Hz	1.01 (0.4)	1.39 (0.37)	1.24 (0.62)	*F* = 4.159 *p* = 0.020	0.016	0.861	0.469
8–12 Hz	2.14 (0.72)	0.95 (0.69)	1.19 (0.94)	*F* = 14.167 *p* ≤ 0.001	<0.001	1.000	0.003
12–30 Hz	0.32 (0.10)	0.15 (0.06)	0.21 (0.09)	*F* = 28.330 *P* ≤ 0.001	<0.001	0.087	<0.001

*HC, healthy controls; EOAD, early-onset Alzheimer disease; FTD, frontotemporal dementia.*

Early-onset Alzheimer’s disease had a higher rPSD in the delta band, compared to FTD. The rPSD of three separated scalp regions (anterior: Fp1, Fp2, F3, F4, C3, C4, Fz, Cz; posterior: P3, P4, O1, O2, Pz; temporal: F7, F8, T3, T4, T5, T6) was calculated and compared among groups. The group comparison results in rPSD of each scalp region were the same with results in global rPSD (Supplementary Material 3).

## Discussion

The current study investigated the EEG microstate’s changes, PSD, and visual EEG in EOAD and FTD. Comparison results between EOAD and LOAD were also presented. The correlations between EEG microstate and clinical severity and CSF biomarkers in AD and FTD were observed.

Cognitive scores and CSF biomarkers were different between FTD and EOAD, as expected. A previous study reported that FTD was associated with greater impairments in ADLs than AD (Mioshi et al., 2007). We found that the two groups had similar ADL scores, but significantly higher MMSE scores in FTD than in EOAD were revealed. It indicated that FTD needs a higher MMSE score to get the same ADL with EOAD. The present study revealed that the ratio of p-tau to Aβ_42_ was significantly increased in EOAD compared to FTD, which is in line with previous studies on AD and FTD (Visser et al., 2009; Vergallo et al., 2017).

The visual EEG severity was correlated with the MMSE score negatively in EOAD and disease course positively in FTD. Previous studies reported the positive correlation between visual EEG scores and clinical severity (Kowalski et al., 2001; de Waal et al., 2011), which was also supported in the present study. We found that visual EEG severity was correlated with CSF biomarkers in FTD. Previous studies observed an inverse correlation between Aβ levels and with the MMSE score (Lewczuk et al., 2020). However, another study also reported that CSF biomarkers had no association with cognition scales (Vemuri et al., 2010). In the current study, we found no correlation between CSF biomarkers and cognition scales, which might be explained with the small sample.

Electroencephalogram microstate topographies in EOAD and FTD significantly deviate from controls. Microstate B and C maps were different between EOAD and control, while the class A map differed between FTD and control. Previous studies on patients with dementias revealed very different results. Two studies revealed no topography differences between AD (mean age 65–70 y) and controls (Stevens and Kircher, 1998; Nishida et al., 2013; Grieder et al., 2016), but topographies of classes B and C in semantic dementia, a variant of FTD, were different from maps in control (Grieder et al., 2016). Schumacher et al. (2019) reported that all five classes (A–E) maps were different between AD (mean age 75 y) and control groups. Another study reported that AD (mean age 68 y) had different topographies of classes A and D compared to the control group (Smailovic et al., 2019). Microstate topography showed poor consistence in all these studies. Age may be one of the factors that influenced the results.

Both FTD and EOAD groups showed increased mean microstate duration and reduced mean occurrence. The increased duration, reduced occurrence, and PPS reflect the loss of microstate dynamics, which may be related to the EEG slowing (Schumacher et al., 2019). Tait et al. also reported that microstate transitions were slower in AD, compared to healthy controls (Tait et al., 2020). Some studies revealed that microstate durations were decreased in patients with dementia or cognitive impairment (Dierks et al., 1997; Strik et al., 1997; Stevens and Kircher, 1998; Nishida et al., 2013). However, more recent studies reported increased durations (Musaeus et al., 2019; Schumacher et al., 2019; Smailovic et al., 2019) and reduced occurrences (Schumacher et al., 2019; Smailovic et al., 2019) in AD, all based on the clustering algorithms, like topographic atomize and agglomerate hierarchical clustering (TAAHC) and k-means. The microstate map classification methods which differed in these studies may be one of the reasons for the different results. We also found that PPS was reduced in AD and FTD, compared to controls. PPS was easier to be calculated, compared to microstate which requires clustering. It showed the same change tendency with mean occurrence, indicating a good simple marker for EEG slowing and microstate dynamics loss.

In addition, microstate variables changes were different in EOAD and FTD. We found increased durations of class B in EOAD and class A in FTD. Microstate C occurrence was decreased in both dementia groups, and microstate A and B occurrences were reduced only in the EOAD group. EOAD had more microstate classes changes, compared to FTD.

Microstate B was significantly different in EOAD for topography and duration and correlated with CSF Aβ_42_ and ADL score, with a high Spearman’s rank coefficient. These class B alterations were not presented in FTD. Previous studies revealed that microstate B was associated with the bilateral occipital cortex (Britz et al., 2010). AD patients have more atrophy in the occipital gyrus and precuneus than FTD patients (Zhang et al., 2011), which may partially explain the difference of microstate B alteration between EOAD and FTD. For microstate A change in FTD, class A was associated with superior and middle temporal lobes (Britz et al., 2010), consistent with the frontotemporal pathologic abnormalities in FTD. These results indicated that microstate may be helpful to differentiate EOAD and FTD.

Cerebrospinal fluid biomarkers were related to microstate in both dementia groups. The Aβ_42_ level was related to microstate B coverage negatively in EOAD and to microstate D occurrence positively in FTD, with high Spearman’s rank coefficient. Smailovic et al. (2019) reported microstate B coverage negatively associated with the Aβ_42_ level in patients with AD (mean age 68 y), but with low Spearman’s rank coefficient. The associations with Aβ_42_ level were also observed in other classes (Smailovic et al., 2019). The current study demonstrated a stronger correlation between microstate and CSF biomarkers in patients with EOAD. CSF Aβ_42_ and tau have high diagnostic accuracy. The correlation between biomarkers and EEG microstate and visual scores indicated that EEG could be a potential diagnostic method for early-onset dementia. Since EEG is a non-invasive and convenient examination, the diagnostic value of microstate for early-onset dementia is worthy of further work.

For EOAD and LOAD group comparisons, earlier studies reported that visual EEG abnormalities were more severe in EOAD (Schreiter-Gasser et al., 1993; de Waal et al., 2011). Since visual EEG results showed no difference between the two AD groups, the microstate differences between EOAD and LOAD indicate that loss of microstate dynamics may be more sensitive than visual EEG slowing. EOAD also showed more microstate changes than LOAD. Moreover, microstate analyses showed that no significant differences in microstate duration and occurrence were observed between LOAD and age-matched controls, which may due to the small sample size. A lager sample study is required.

The spectral analysis demonstrated that FTD and EOAD had lower rPSD in alpha and beta bands, and higher rPSD in delta bands, indicating that the general EEG was slowing. It further suggests that loss of microstate dynamics may be attributed to EEG slowing. A diffuse slowing with reduction of power in faster rhythm and increased power in slow rhythm has been observed in AD (Bennys et al., 2001; Malek et al., 2017) and FTD (Lindau et al., 2003). Additionally, the power in the delta band was increased in AD compared to FTD (Lindau et al., 2003; Caso et al., 2012), which was consistent with findings in the current study. We found delta band rPSD in EOAD was higher than rPSD in FTD all over the head, indicating a more diffuse slowing in EOAD than FTD.

## Limitations

The present study has some limitations. First, the sample size of EOAD and FTD patients with CSF biomarker results was small. Therefore, the correlation analysis results with low Spearman’s rank coefficient and significance level were not strong enough. A larger sample will draw more convincing conclusions. Second, the sample size of LOAD was small, and patients with late-onset FTD (>65 y) were lacked. In addition, part of patients with dementia were taking AChEIs which may influence EEG data (Babiloni et al., 2013). There was no difference in the number of patients taking AChEIs between FTD and EOAD. However, group comparisons between dementia and control groups may be influenced by the use of AChEIs. Further work, like better statistical analysis, or the set of prospective studies, may help to solve the effect.

## Conclusion

The current study demonstrated that EOAD and FTD both had EEG slowing and loss of microstate dynamics, compared to controls. Moreover, EOAD and FTD had different microstate class changes, with no differences in visual EEG results. Similar results were also observed in-group comparisons between EOAD and LOAD. It indicated that microstate is more sensitive than visual EEG and may be useful for differentiation between EOAD and FTD. Correlations with clinical severity and CSF biomarkers were observed in EOAD and FTD, suggesting that microstate could be a potential marker for dementia diagnosis and clinical severity evaluations. However, microstate analyses can produce numerous variables. Some variables, like topography, had poor consistency, while some variables, like durations, which were not specific to one class, showed similar characteristics in recent studies. Age and clustering methods may be the reasons, but more work is required to identify which EEG variables are useful for disease diagnosis and evaluations.

## Data Availability Statement

The original contributions presented in the study are included in the article/Supplementary Material, further inquiries can be directed to the corresponding author/s.

## Ethics Statement

The studies involving human participants were reviewed and approved by The Ethics Committee of Peking Union Medical College Hospital (No. JS-2089). The patients/participants provided their written informed consent to participate in this study.

## Author Contributions

NL analyzed and interpreted the data and wrote the original manuscript. JG analyzed the data, diagnosed the patients, and revised the manuscript. QL and LC conceived the study and revised the manuscript. CM acquired the data and diagnosed the patients. HS performed the EEG recordings and analyzed visual EEG data. All authors read and approved the final manuscript. All authors agreed to be accountable for the content of the work.

## Conflict of Interest

The authors declare that the research was conducted in the absence of any commercial or financial relationships that could be construed as a potential conflict of interest.

## Publisher’s Note

All claims expressed in this article are solely those of the authors and do not necessarily represent those of their affiliated organizations, or those of the publisher, the editors and the reviewers. Any product that may be evaluated in this article, or claim that may be made by its manufacturer, is not guaranteed or endorsed by the publisher.
