# Supplementary material for: Differences in Multimodal Electroencephalogram and Clinical Correlations Between Early-Onset Alzheimer’s Disease and Frontotemporal Dementia
Source: Front Neurosci. 2021 Aug 5;15:687053. doi: 10.3389/fnins.2021.687053 (PMC8374312; doi:10.3389/fnins.2021.687053)
Supplement: Supplementary file 1 [file Data_Sheet_1.docx]

Supplementary Material

**
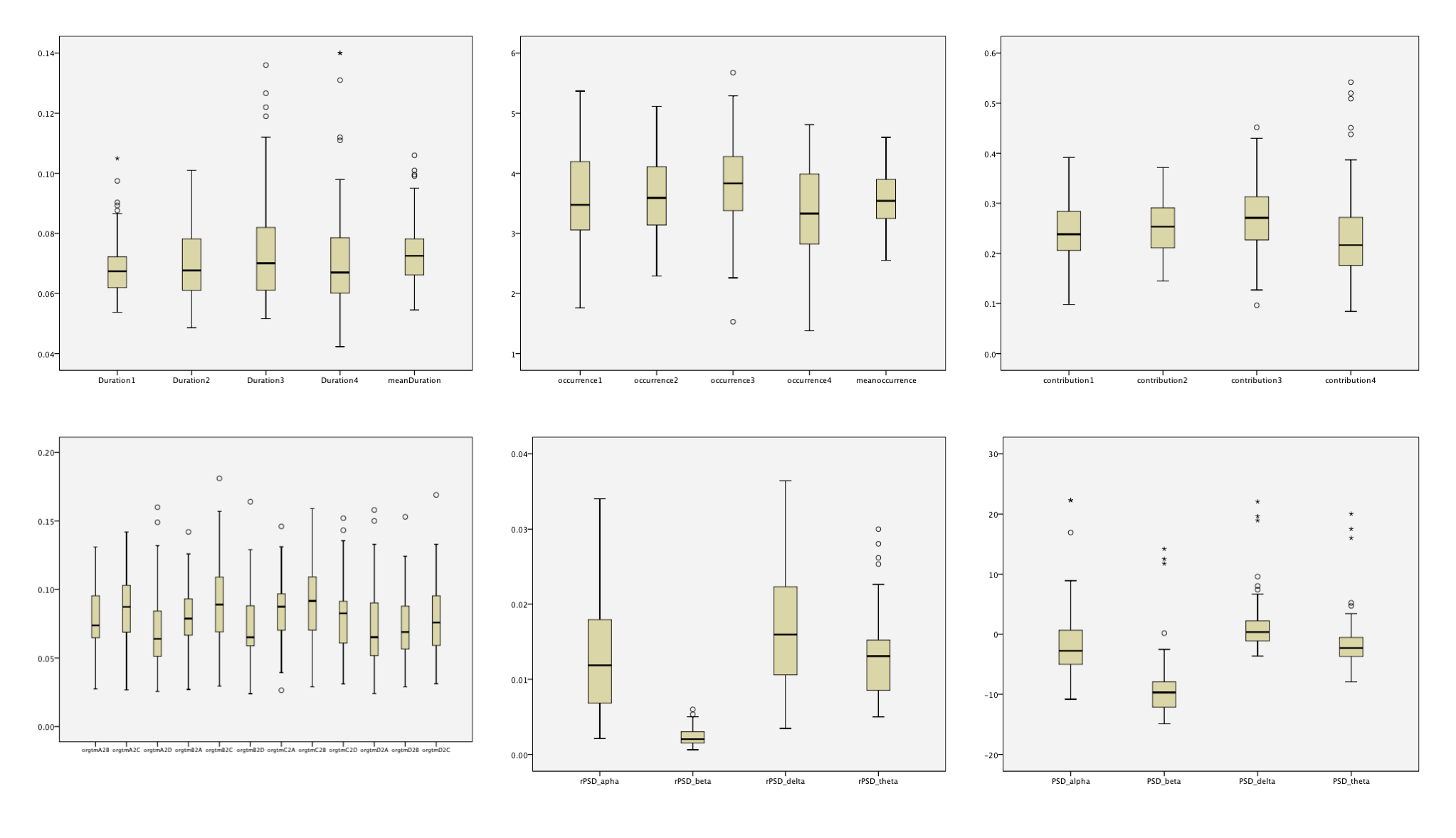
**

**Supplementary 1.** Boxplots of microstate and spectral variables. Data distributions were relatively symmetrical in terms of microstate duration (A), occurrence (B), coverage (C), transition probability (D), absolute PSD (E) and relative PSD (F) in each frequency band. The normal assumption was intuitively conformed. PSD: power spectral density.

**Supplementary 2.** Microstate coverage and transition probability in dementia and control groups

| Coverage (Std) | HC | EOAD | FTD | ANOVA  (2,59) |
| --- | --- | --- | --- | --- |
| A | 0.25 (0.06) | 0.22 (0.04) | 0.26(0.067) | F=3.096 P=0.053 |
| B | 0.25 (0.07) | 0.25 (0.04) | 0.24(0.061) | F=0.132 P=0.876 |
| C | 0.28 (0.07) | 0.28 (0.07) | 0.24(0.072) | F=1.412 P=0.252 |
| D | 0.22 (0.08) | 0.25 (0.11) | 0.25(0.12) | F=0.672 P=0.515 |
| Transition probability (Std) | HC | EOAD | FTD | ANOVA  (2,59) |
| A->B | 0.077(0.019) | 0.071(0.019) | 0.087(0.026) | F=2.822 P=0.068 |
| A->C | 0.094(0.022) | 0.081(0.023) | 0.079(0.027) | F=2.070 P=0.135 |
| A->D | 0.066(0.027) | 0.071(0.030) | 0.077(0.034) | F=0.522 P=0.596 |
| B->A | 0.078(0.020) | 0.073(0.018) | 0.087(0.030) | F=1.821 P=0.171 |
| B->C | 0.096(0.033) | 0.092(0.031) | 0.080(0.032) | F=0.993 P=0.377 |
| B->D | 0.069(0.023) | 0.074(0.027) | 0.077(0.025) | F=0.360 P=0.699 |
| C->A | 0.090(0.019) | 0.080(0.021) | 0.082(0.027) | F=1.074 P=0.348 |
| C->B | 0.097(0.032) | 0.094(0.029) | 0.079(0.029) | F=1.637 P=0.203 |
| C->D | 0.080(0.023) | 0.085(0.023) | 0.077(0.029) | F=0.561 P=0.574 |
| D->A | 0.067(0.028) | 0.072(0.029) | 0.077(0.034) | F=0.430 P=0.653 |
| D->B | 0.069(0.026) | 0.075(0.026) | 0.076(0.022) | F=0.391 P=0.678 |
| D->C | 0.078(0.020) | 0.086(0.021) | 0.078(0.035) | F=0.765 P=0.470 |

HC: healthy controls. EOAD: early onset Alzheimer disease; FTD: frontotemporal dementia.

**Supplementary 3.** Power spectral density in dementia and control groups

| rPSD (%, mean, std) | Region | HC | EOAD | FTD | MANOVA  (d=8) | P_HC-EOAD_ | P_EOAD-FTD_ | P_HC-FTD_ |
| --- | --- | --- | --- | --- | --- | --- | --- | --- |
| 1-4Hz | Anterior | 1.06 (0.41) | 2.28 (0.56) | 1.80 (0.78) | <0.001 | <0.001 | 0.041 | 0.002 |
|  | Posterior | 0.78 (0.35) | 2.06(0.62) | 1.52 (0.98) | <0.001 | <0.001 | 0.043 | 0.008 |
|  | Temporal | 1.05 (0.37) | 2.22(0.50) | 1.71 (0.71) | <0.001 | <0.001 | 0.011 | 0.003 |
| 4-8Hz | Anterior | 1.00(0.40) | 1.34 (0.36) | 1.21 (0.53) | 0.027 | 0.022 | 0.973 | 0.487 |
|  | Posterior | 0.98 (0.50) | 1.45 (0.47) | 1.27 (0.80) | 0.027 | 0.022 | 0.990 | 0.476 |
|  | Temporal | 1.03 (0.38) | 1.41 (0.36) | 1.24 (0.60) | 0.018 | 0.015 | 0.708 | 0.536 |
| 8-12Hz | Anterior | 1.99 (0.73) | 0.88 (0.69) | 1.06 (0.82) | <0.001 | <0.001 | 1.000 | 0.002 |
|  | Posterior | 2.59 (0.86) | 1.17 (0.86) | 1.50 (1.27) | <0.001 | <0.001 | 0.875 | 0.007 |
|  | Temporal | 1.97 (0.64) | 0.87 (0.60) | 1.09 (0.85) | <0.001 | <0.001 | 0.901 | 0.002 |
| 12-30Hz | Anterior | 0.34 (0.12) | 0.16 (0.06) | 0.21 (0.09) | <0.001 | <0.001 | 0.155 | 0.001 |
|  | Posterior | 0.31 (0.11) | 0.15 (0.06) | 0.20 (0.10) | <0.001 | <0.001 | 0.206 | 0.003 |
|  | Temporal | 0.34 (0.09) | 0.15 (0.07) | 0.22 (0.10) | <0.001 | <0.001 | 0.052 | <0.001 |

Anterior: Fp1, Fp2, F3, F4, C3, C4, Fz, Cz; Posterior: P3, P4, O1, O2, Pz; Temporal: F7, F8, T3, T4, T5, T6. HC: healthy controls. EOAD: early onset Alzheimer disease; FTD: frontotemporal dementia.
