# Supplementary figures and images for: Differences in Multimodal Electroencephalogram and Clinical Correlations Between Early-Onset Alzheimer’s Disease and Frontotemporal Dementia
Source: Front Neurosci. 2021 Aug 5;15:687053. doi: 10.3389/fnins.2021.687053 (PMC8374312; doi:10.3389/fnins.2021.687053)

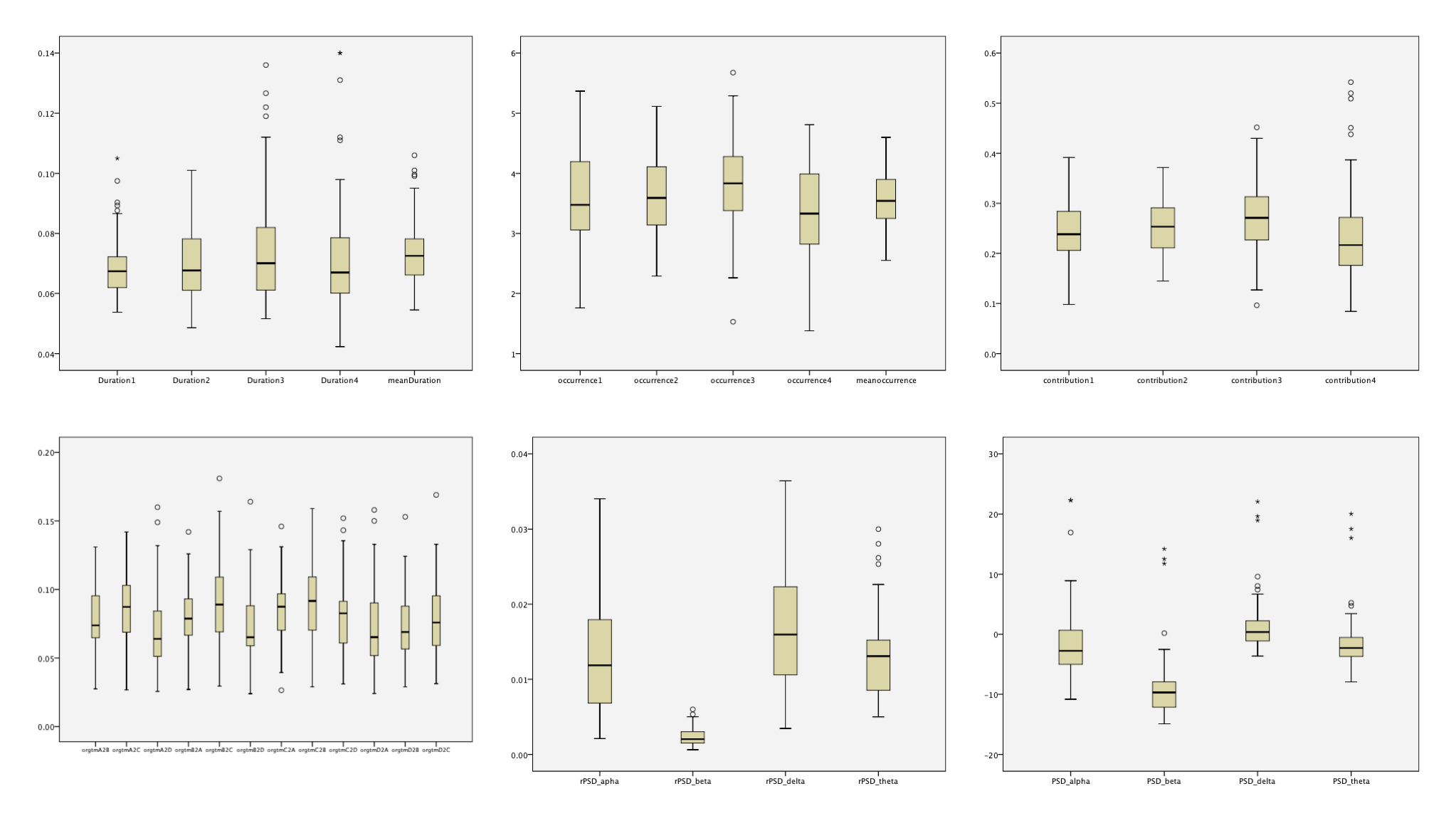

Supplement: Supplementary file 2 [file Image_1.TIFF]
